# Largemouth Bass Virus Infection Induced Non-Apoptotic Cell Death in MsF Cells

**DOI:** 10.3390/v14071568

**Published:** 2022-07-19

**Authors:** Jiahui Yang, Weihua Xu, Wenji Wang, Zanbin Pan, Qiwei Qin, Xiaohong Huang, Youhua Huang

**Affiliations:** 1 Laboratory for Lingnan Modern Agriculture, College of Marine Sciences, South China Agricultural University, Guangzhou 510642, China; yjhaijy@gmail.com (J.Y.); xuweihua@stu.scau.edu.cn (W.X.); wangwenji653750@stu.scau.edu.cn (W.W.); pzanbin@gmail.com (Z.P.); qinqw@scau.edu.cn (Q.Q.); 2 University Joint Laboratory of Guangdong Province, Hong Kong and Macao Region on Marine Bioresource Conservation and Exploitation, Guangzhou 510642, China

**Keywords:** ranavirus, LMBV, non-apoptotic cell death, mitochondrion, ROS

## Abstract

Largemouth bass virus (LMBV), belonging to the genus *Ranavirus*, causes high mortality and heavy economic losses in largemouth bass aquaculture. In the present study, a novel cell line, designated as MsF, was established from the fin of largemouth bass (*Micropterus salmoides*), and applied to investigate the characteristics of cell death induced by LMBV. MsF cells showed susceptibility to LMBV, evidenced by the occurrence of a cytopathic effect (CPE), increased viral gene transcription, protein synthesis, and viral titers. In LMBV-infected MsF cells, two or more virus assembly sites were observed around the nucleus. Notably, no apoptotic bodies occurred in LMBV-infected MsF cells after nucleus staining, suggesting that cell death induced by LMBV in host cells was distinct from apoptosis. Consistently, DNA fragmentation was not detected in LMBV-infected MsF cells. Furthermore, only caspase-8 and caspase-3 were significantly activated in LMBV-infected MsF cells, suggesting that caspases were involved in non-apoptotic cell death induced by LMBV in host cells. In addition, the disruption of the mitochondrial membrane potential (ΔΨm) and reactive oxygen species (ROS) generation were detected in both LMBV-infected MsF cells and fathead minnow (FHM) cells. Combined with our previous study, we propose that cell death induced by LMBV infection was cell type dependent. Although LMBV-infected MsF cells showed the characteristics of non-apoptotic cell death, the signal pathways might crosstalk and interconnect between apoptosis and other PCD during LMBV infection. Together, our results not only established the in vitro LMBV infection model for the study of the interaction between LMBV and host cells but also shed new insights into the mechanisms of ranavirus pathogenesis.

## 1. Introduction

As intracellular pathogens, viruses require host cell machinery to complete their life cycle. On the contrary, the host has developed a variety of mechanisms to initiate an immune response to clear the virus. Programmed cell death (PCD), a key component of the innate immune response, is not only an effective strategy for the host cell to restrict virus infection [1] but also plays an important role in the pathogenesis of viral diseases [2]. To date, the most well-defined and studied PCD pathways include apoptosis, autophagy, necroptosis, and pyroptosis. Numerous pieces of evidence have demonstrated that many DNA or RNA viruses induce the activation of one or more cell death pathways [2,3]. For example, Dengue virus infection induces pyroptosis in macrophages and dendritic cells [4]. Respiratory syncytial virus (RSV) infection promotes necroptosis in airway epithelia cells (AECs) [5]. Apoptosis is the most extensively studied PCD in viral infection. For many viruses, including human immunodeficiency virus (HIV) [6], hepatitis virus (HCV) [7], varicella-zoster virus (VZV) [8], influenza virus [9], and West Nile virus (WNV) [10], induction of apoptosis may be an important way to release and disseminate progeny viruses [11]. Recently, a highly interconnected PCD called PANoptosis was defined, and PANoptosis has been involved in microbial infections and in cancer and autoinflammatory diseases [12,13,14].

Iridoviruses are large DNA viruses that cause global amphibian declines and heavy economic losses in freshwater and marine aquaculture annually [15,16,17]. Members of the family *Iridoviridae* are now classified into two subfamilies: Alphairidovirinae and Betairidovirinae. To date, members in three genera, including *Megalocytivirus*, *Ranavirus*, and *Lymphocystivirus*, which belongs to Alphairidovirinae, are able to infect lower vertebrates. Among them, Ranaviruses have a broad host range, including teleost, amphibians, and reptiles [18]. To demonstrate the mechanism of ranaviruses pathogenesis, the mode of cell death induced by ranaviruses was gradually investigated, including Rana grylio virus (RGV) [19], Frog virus 3 (FV3) [20], grouper iridovirus (GIV) [21], epizootic hematopoietic necrosis virus (EHNV) [22], and Chinese giant salamander iridovirus (GSIV) [23]. Interestingly, the outcome of cell death induced by SGIV (fish ranavirus) was cell type dependent [24]. In host cells (grouper cells), SGIV infection induced nonapoptotic PCD while the infection in non-host cells showed typical biochemical features of apoptosis [24,25]. However, whether more evidence supports the conclusion that ranaviruses modulate the survival of host and non-host cells differently requires further study [21].

Largemouth bass (*Micropterus salmoides*), an important freshwater farmed species, has been widely cultured in China in recent years [26,27]. However, the emergence of viral diseases has caused great economic losses in largemouth bass aquaculture. Largemouth bass virus (LMBV), belonging to the genus *Ranavirus*, is the important causative agent, which causes high mortality rates over 60% in largemouth bass [28,29,30]. LMBV was successfully propagated and isolated in epithelioma papulosum cyprini (EPC) and fathead minnow (FHM) cells. A previous study showed that LMBV induced typical apoptosis in EPC cells [31]. Recently, the literature revealed that autophagy induced by LMBV suppressed virus replication and blocked apoptosis in EPC cells [32]. However, limited information is focused on the cell death induced by LMBV in host cells.

In the present study, a new cell line, designated as MsF, was established from the fin of largemouth bass. An in vitro LMBV infection model was developed using MsF cells and the characteristics of cell death induced by LMBV infection in this cell line were investigated. Our results not only provide a useful tool for studying the interaction between LMBV and host cells but also shed new insights on the understanding of the mechanisms of ranavirus pathogenesis.

## 2. Materials and Methods

### 2.1. Primary Cell Culture and Subculture

Largemouth bass with a size of 7 cm were obtained from a local farm in Foshan city, Guangdong province, and were used for primary cell culture as described previously [33]. In detail, fish were anesthetized and then wiped with 70% alcohol. The caudal fin was cut off aseptically and washed three times in an antibiotic medium (Leibovitz’s L-15 with 400 IU/mL penicillin, 400 µg/mL streptomycin, and 400 µg/mL nystatin). The caudal fin was cut into small pieces approximately 2 × 2 mm in size. After washing three times with medium containing antibiotics, the small pieces were transferred to the bottom of a flask for 1 h. Then, L-15 medium containing antibiotics and 20% fetal bovine serum (FBS) were added, and placed in an incubator for further culture at 28 °C.

Once a confluent monolayer formed in primary culture, cells were trypsinized with 0.25% trypsin-EDTA solution (Invitrogen, Waltham, MA, USA) and subcultured as described previously [33]. In brief, the primary cells were subcultured at a 1:2 ratio and maintained in L-15 medium containing 20% FBS and antibiotics (400 IU/mL penicillin, 400 µg/mL streptomycin, and 400 µg/mL nystatin). The cells were subcultured every 3–4 days.

### 2.2. Viral Replication Dynamics

To assess the susceptibility of MsF cells to LMBV, MsF cells were seeded into a 24-well plate overnight, and then infected with LMBV for several blind passages. The appearance of a cytopathic effect (CPE) induced by LMBV passage 3 was observed daily for up to 3 days and photographed under a Zeiss microscope.

To further evaluate the replication of LMBV in MsF cells, cells were infected with LMBV at an MOI of 2 and harvested for the preparation of ultrathin sections as described previously [33]. In brief, after prefixation with 2.5% glutaraldehyde overnight, the cell pellets were post-fixed in 1% osmium tetroxide for 1 h and then dehydrated in graded ethanol. After Epon resin embedding, samples were sectioned and double stained with uranyl acetate and lead citrate. The grids containing ultrathin sections were observed under a Talos L120C transmission electron microscope (TEM) (Thermo Fisher Scientific, Waltham, MA, USA) at 120 KV.

### 2.3. RNA Extraction and Quantitative Polymerase Chain Reaction (qPCR)

At the indicated time points (12, 24, 36, and 48 h postinfection), LMBV-infected cells were harvested for RNA extraction as described previously [33]. In brief, the total RNA of infected cells was extracted using a cell total RNA isolation kit according to the manufacturer’s protocol. The RNA was reverse transcribed using a ReverTra Ace-a kit (Toyobo, Osaka, Japan). The transcription level of viral genes, including major capsid protein (MCP), myristoylated membrane protein (MMP), and DNA methytransferase (DNMT), were examined using qPCR analysis. qPCR was performed using SYBR^®^ Green Realtime PCR Master Mix (Toyobo, Osaka, Japan) in an Applied Biosystems QuantStudio 5 Real Time detection system (Thermo Fisher Scientific) as described previously. The cycling conditions for qPCR were as follows: 95 °C for 5 min for activation followed by 45 cycles at 95 °C for 5 s, 60 °C for 10 s, and 72 °C for 15 s. The primers used in this study are listed in Table 1. All samples were carried out in triplicate, and the expression level of target genes was normalized to β-actin and calculated using the 2^−ΔΔCT^ method. The data are presented as mean ± SD. Statistics were calculated using SPSS version 20 by one-way ANOVA. Differences were considered to be statistically significant when *p* < 0.05 (*).

### 2.4. Western Blotting

At the indicated time points (12, 24, 36, and 48 h p.i.), mock- and LMBV-infected cells were harvested for Western blotting assay as described previously [24]. In brief, the pellets of cells were lysed and solubilized in Pierce IP lysis buffer (Thermo Fisher Scientific), containing protease/phosphatase inhibitor cocktail. After boiling for 5 min, the equal of proteins was resolved by 10% sodium dodecyl sulfatepolyacrylmide gel electrophoresis (SDS-PAGE), and then transferred to a PVDF membrane (Millipore). The membranes were blocked with 5% skim milk for 2 h, and then incubated with the antibody against LMBV MCP (1:1500 dilution) and anti-β-tubulin (Abcam, Cambridge, UK; 1:1000 dilution) for 2 h at room temperature, respectively. After washing with TBST, the membrane was then incubated with horseradish peroxidase (HRP)-conjugated sheep-mouse IgG at a dilution of 1:5000 (Abcam) for 2 h at room temperature. The immunoblot bands were visualized using an enhanced HRP-DAB Substrate Chromogenic Kit (Tiangen, Sichuan, China) according to the manufacturer’s protocol. The experiments were independently carried out three times. Due to highly similar data, the results presented are from one representative experiment.

### 2.5. Immunofluorescence Assay (IFA)

At the indicated time points (12, 24, 36, and 48 h p.i.), mock- and LMBV-infected cells were harvested for immunofluorescence assay as described previously [24]. In brief, MsF cells were seeded into 35-mm glass-bottom cell culture dishes overnight, and then infected with LMBV at an MOI of 2. After fixation with 4% paraformaldehyde, the cells were permeabilized with 0.2% triton X-100 for 10 min followed by blocking with 2% bovine serum albumin. The cells were incubated with mouse anti-LMBV MCP serum (1:500) for 2 h and then incubated with secondary antibody anti-mouse IgG Fab2 Alexa Fluor 555 (1:500, Molecular probe). Finally, the cells were stained with 1 μg/mL 4′,6- diamidino-2-phenylindole (DAPI) (Sigma, St. Louis, MI, USA) and observed under an inverted fluorescence microscope.

### 2.6. Virus Titer Assay

The viral titer in the cell lysates was assessed on monolayers of MsF cells using the 50% tissue culture infectious dose (TCID_50_) assay. At the indicated time points (12, 24, 36, and 48 h p.i.), LMBV-infected cells were collected for virus titer assay. Adherent cells containing medium were frozen and thawed three times (lysate). Samples were serially diluted 10-fold, overlaid on 95% confluent monolayers of MsF cells in 96-well plates, and incubated for 1 h. After removing the non-adsorbed virus, fresh medium was added to the wells, and the cells were incubated at 28 °C for 6 days. The cytopathic effects were observed under a light microscope (Leica, Wetzlar, Germany) every day. The virus titration of each sample was measured in triplicate, and the data are presented as the means ± standard deviation (SD).

### 2.7. TUNEL Assay

To detect DNA fragmentation in situ, the terminal deoxynucleotidyl transferase-mediated nicked-end labeling (TUNEL) assay was carried out as described previously [24]. MsF or FHM cells were seeded into a 24-well plate overnight, and then infected with LMBV at an MOI of 2. Mock- or LMBV-infected cells were fixed with 4% paraformaldehyde at 48 h p.i. After washing with PBS, the cells were stained with TUNEL reaction buffer. The emitted green fluorescence of the apoptotic cells was observed under a fluorescence microscope (Zeiss, Oberkochen, Germany).

### 2.8. Flow Cytometric Analysis

The proportion of apoptotic cells (cells in the sub-G0/G1 cell cycle fraction) were determined by flow cytometry analysis as described previously [24]. In brief, mock- and virus-infected cells were collected and fixed in 70% ice-cold ethanol overnight at −20 °C. After washing with PBS, the cells were centrifuged at 1000 rpm/min for 10 min. The pellets were resuspended in PBS containing 50 μg/mL propidium iodide (PI, Sigma) and 50 μg/mL DNase-free RNase A, and stained for 30 min. The PI fluorescence was measured with a Beckman Coulter flow cytometer (Brea, CA, USA), and 10^4^ cells were analyzed for each sample, and each sample was measured in triplicate. The obtained data were analyzed using FlowJo 10.4 software, and the results presented were from one representative experiment.

### 2.9. Detection of Caspase Activities

At the indicated time points (12, 18, 24, 36, and 48 h p.i.), mock- and LMBV-infected cells were harvested for the detection of caspase activities. The activities of caspase-3, caspase-8, or caspase-9 were detected using a caspase-3, caspase-8, and caspase-9 multiplex activity assay kit (Abcam) according to the protocol booklet supplied by the manufacturer. Briefly, MsF cells inoculated into 96-well plates were incubated with LMBV at the indicated time points, respectively. Untreated cells were used as a control. Then, loading solution (mixture of Assay Buffer and caspase-3 substrate, caspase-8 substrate, and caspase-9 substrate) was added to react for 30 min at room temperature in the dark. The caspase activities were monitored in a fluorescence microplate reader at the specific wavelengths (Casp3: Ex/Em = 535/620 nm, Casp8: Ex/Em = 490/525 nm, and Casp9: Ex/Em = 370/450 nm). Caspase activation was evaluated as fold increases by comparing the readings obtained from treated cells with measurements from control cells. Independent experiments were performed in triplicate.

### 2.10. Evaluation of the Mitochondrial Membrane Potential (ΔΨm)

At the indicated time points (12, 24, 36, and 48 h p.i.), mock- and LMBV-infected cells were harvested for the detection of ΔΨm as described previously [24]. The cells were washed with the fresh medium, and stained with buffer containing 50 μg/mL JC-1 dye (Thermo Fisher Scientific) for 20 min. After washing with fresh medium, the fresh medium was added into wells. The fluorescence of the cells was examined under fluorescent microscopy.

In order to quantify the percentages of cells with decreased ΔΨm, after staining with JC-1, mock- and LMBV-infected cells were harvested for fluorescence spectrometry using a fluorescence microplate reader. A 488-nm filter was used for excitation of JC-1 and the emissions at 535 and 595 nm were used to quantify the population of mitochondria with green (JC-1 monomers) and red (JC-1 aggregates) fluorescence, respectively. The red/green ratio was used to reflect the mitochondrial membrane potential.

### 2.11. Reactive Oxygen Species (ROS) Activity

In order to examine the ROS generation during LMBV infection, the redox-sensitive fluorescent probe CM-H2DCFDA was used for ROS analysis as described previously [24]. MsF or FHM cells were seeded onto a 24-well plate overnight, and then infected with LMBV at an MOI of 2. At 24 and 48 h p.i., mock- and virus-infected cells were washed with the fresh medium, and then incubated with medium containing 10 μM CM-H2DCFDA for 1 h. After washing with fresh medium, the fresh medium was added into the wells, and the samples were observed under a fluorescence microscope.

### 2.12. Statistical Analysis

The statistical analyses were performed using SPSS version 20. A one-way ANOVA was used to evaluate the differences between groups. Differences were considered statistically significant when the *p*-value was < 0.05.

## 3. Results

### 3.1. Primary Culture and Subculture

**Figure 1:**
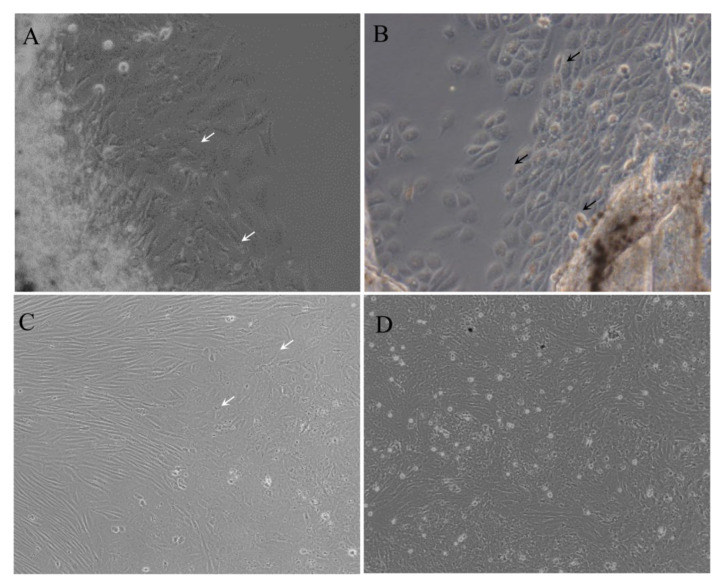
Morphology of MsF cells at different passages. Cells at primary culture (**A**,**B**), passage 10 (**C**), and passage 50 (**D**) were photographed, respectively. Black and white arrows indicate the epithelial-like and fibroblast-like cells, respectively.

The cells were migrated from the fin tissue pieces on day 5. Morphologically, the primary culture cells consisted of both epithelial-like and fibroblast-like cells. A confluent monolayer formed on about day 25. The cells were subcultured in L-15 medium with 20% FBS at a ratio of 1:2 every 3–4 days. To date, the cells were subcultured for more than 120 passages, and this was designated as the MsF cell line. After 60 subculture passages, MsF cell lines were predominantly composed of epithelial-like cells (Figure 1).

### 3.2. Establishment of the In Vitro LMBV Infection Model

**Figure 2:**
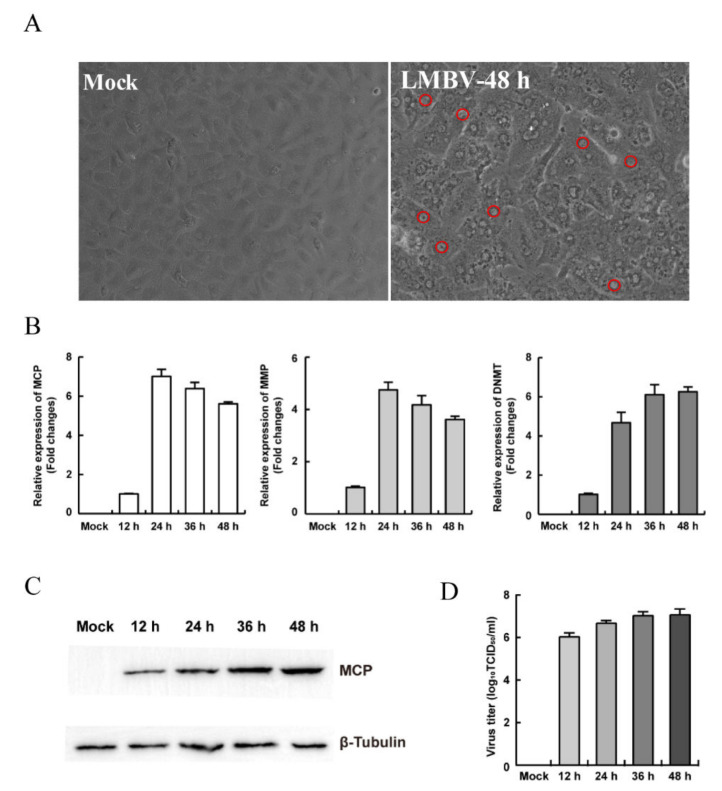
The susceptibility of MsF cells to fish viruses. (**A**) The cytopathic effects of MsF cells induced by LMBV. The red circles indicate the “vacuole”-like structures in LMBV-infected cells. (**B**) The transcription levels of viral genes in LMBV-infected MsF cells. (**C**) The protein synthesis of MCP in LMBV-infected MsF cells. (**D**) Virus production in LMBV-infected MsF cells at different infection time.

To evaluate the susceptibility of MsF cells to virus, LMBV was incubated with MsF cells and collected for the first blind passage. After three blind passages, the CPE progression of LMBV-infected cells was observed under phase contrast microscopy. As shown in Figure 2A, no obviously rounded cells occurred at 48 h after LMBV infection while numerous “vacuole”-like structures were observed in the cytoplasm in LMBV-infected MsF cells compared with mock-infected cells. In contrast, no “vacuole”-like structures were observed in mock-infected cells at 48 h p.i. (Figure 2A).

In order to determine whether LMBV could successfully replicate in MsF cells, viral gene transcription and expression during LMBV infection was detected by qPCR and Western blotting. As shown in Figure 2B, the transcription levels of LMBV MCP and MMP were gradually increased up to 24 h p.i., and then slowly decreased, whereas DMNT transcription was gradually increased with the time of infection. Similarly, the Western blotting result showed that the expression of LMBV MCP was gradually increased in MsF-infected cells (Figure 2C). Furthermore, the virus production in LMBV-infected cells was determined by virus titer assay. The viral titer increased significantly with infection time, and the titer increased significantly from 12 to 48 h p.i. (Figure 2D). Taken together, the results indicate that LMBV successfully replicated in MsF cells, and the in vitro LMBV infection model is a good potential tool for study of the virus–host cell interaction.

### 3.3. The Characteristics of Replication and Assembly of LMBV in MsF Cells

**Figure 3:**
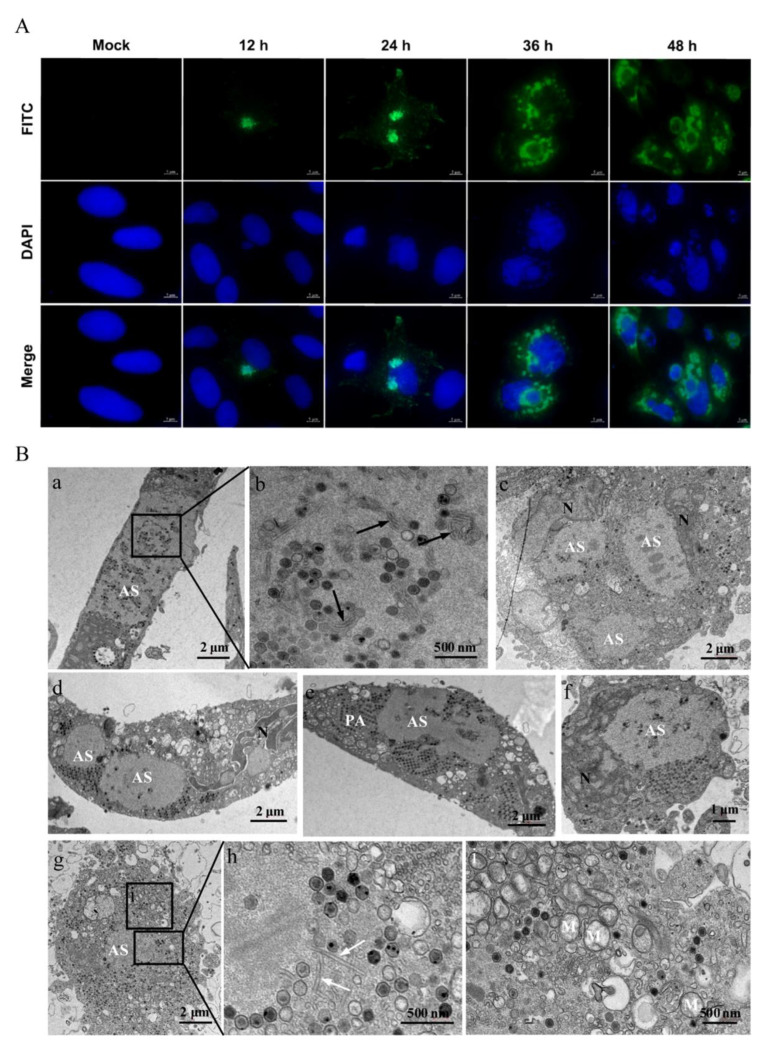
The characteristics of LMBV replication in MsF cells. (**A**) The intracellular localization of viral MCP in LMBV-infected MsF cells was detected using immunofluorescence assay. (**B**) The ultrastructural characteristics of LMBV assembly in MsF cells. (**a**,**g**): Numerous viral particles were observed in assembly sites (ASs) in LMBV-infected MsF cells. (**b**,**h**): Some amorphous or tubular structures were observed in assembly sites. Black and white arrows showed the amorphous and tubular structures, respectively. (**c**–**f**): The numerous hexagonal virus particles formed paracrystalline arrays (PAs) or scattered around the assembly sites in LMBV-infected MsF cells. (**i**): The vacuolized mitochondria were observed around the assembly sites. M: mitochondria; N: nucleus.

In order to detect the characteristics of viral replication in LMBV-infected MsF cells, the synthesis and localization of LMBV MCP protein were determined using immunofluorescence assay. As shown in Figure 3A, a small viral assembly site was observed in partly infected cells at 12 h p.i., and the green fluorescence signal of MCP was mainly located at the assembly sites. The numbers of assembly sites increased in some infected cells at 24 h p.i., and punctuated green fluorescence signals were also observed in the cytoplasm. With the extension of the infection time, although the fluorescence signal of MCP in the cytoplasm was significantly enhanced, the signals in the virus assembly site were decreased at 36 h p.i. At the late stage of infection, the intensive green fluorescence was mainly distributed around the assembly sites.

Furthermore, the ultrastructure of LMBV-infected MsF cells was observed by electron microscopy. As shown in Figure 3B, a large number of virus particles was observed at the assembly sites (Figure 3B(a,g)). In addition to viral particles, some amorphous tubular structures with an ~20-nm diameter and different stages of virus particles were also observed at the assembly sites (Figure 3B(b,h)). Of note, two or more assembly sites were observed in some infected cells (Figure 3B(c,d)) and two adjacent assembly sites were fused into a large one (Figure 3B(e)). At the later stage of infection, numerous hexagonal virus particles formed paracrystalline arrays or scattered around the assembly sites in LMBV-infected cells (Figure 3B(c–f)). The nuclear morphology of the infected cells was severely deformed, and the chromatin of the nuclei was condensed and marginated (Figure 3B(c,d,f)). In addition, the cristae of mitochondria were faint or disappeared, and vacuolized mitochondria were observed around the assembly sites (Figure 3B(i)).

### 3.4. LMBV Infection Induced Nonapoptotic Cell Death in MsF Cells

**Figure 4:**
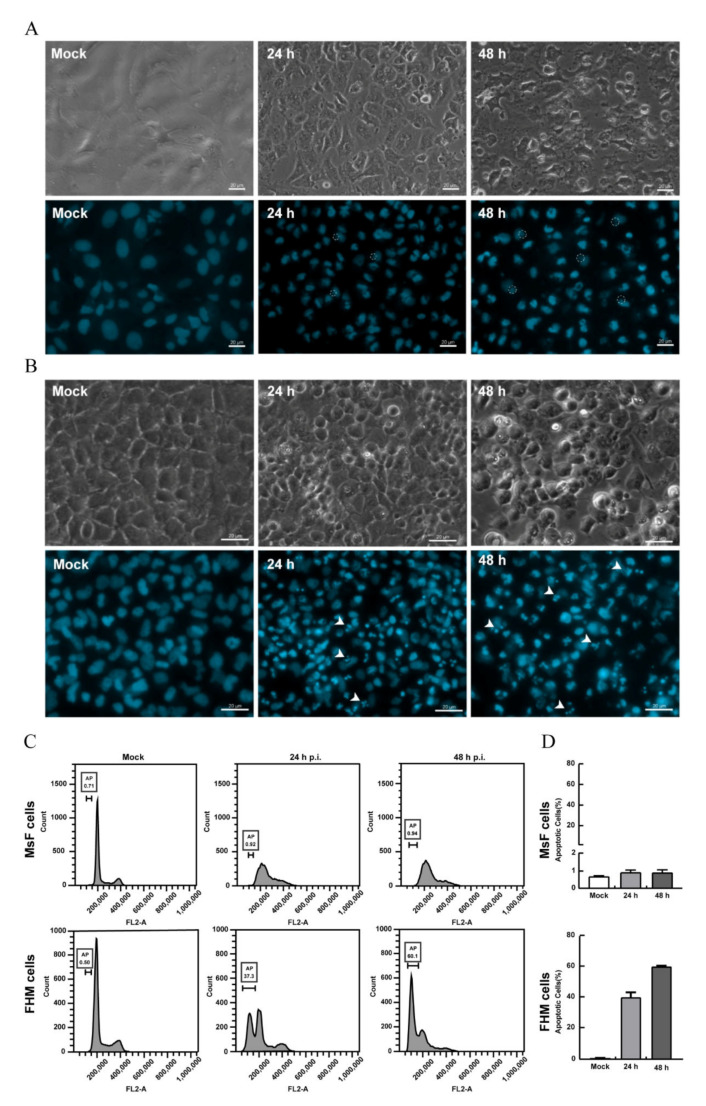
LMBV infection induced non-apoptotic cell death in MsF cells. (**A**) The nuclear morphology of mock- or LMBV-infected MsF cells. Few apoptotic bodies were observed in LMBV-infected MsF cells. Circles indicate the assembly sites. (**B**) The nuclear morphology of mock- or LMBV-infected FHM cells. Numerous apoptotic bodies were observed in LMBV-infected FHM cells. Head arrows indicate apoptotic bodies. (**C**) DNA content analysis in LMBV-infected MsF and FHM cells. (**D**) The percentages of the sub-G0/G1 phase (apoptotic cells) in LMBV-infected MsF and FHM cells.

**Figure 5:**
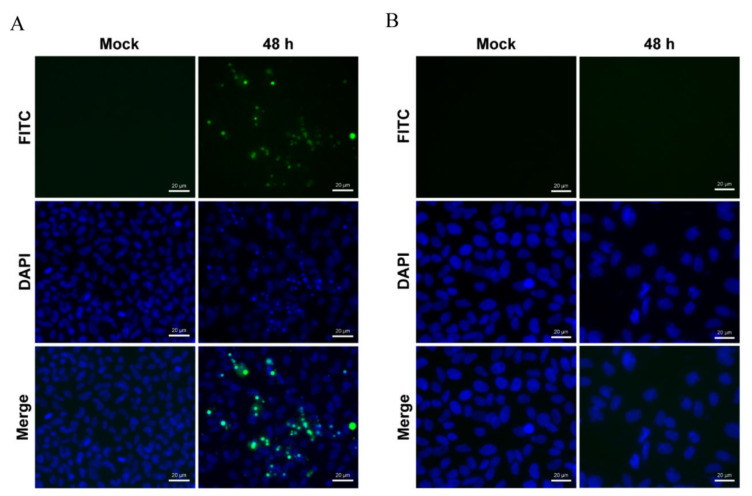
TUNEL staining of LMBV-infected cells. LMBV infected FHM (**A**) and MsF (**B**) cells were stained with TUNEL and DAPI, and the fluorescence was observed under fluorescence microscopy.

Our previous study showed that LMBV infection in EPC cells induced typical apoptosis [31]. Given the obvious difference in CPE induced by LMBV in host cells (MsF cells) and non-host cells (EPC cells), it is speculated that the cell death induced by LMBV infection in MsF cells was different from typical apoptosis. The biochemical features of cell death induced by LMBV in MsF cells were investigated by fluorescent microscopy and flow cytometry analysis. The nuclear morphological changes during LMBV infection were determined after staining with DAPI. As shown in Figure 4B, in LMBV-infected FHM cells, apoptotic bodies were observed at 24 h p.i., and a large number of cells underwent nuclear condensation and fragmentation. With the infection time, increasingly more cells became rounded, which was accompanied by an increase in apoptotic bodies. Differently, although the morphology of nuclei was abnormal, taking the shape of a crescent, and viral assembly sites were observed adjacent to the nucleus in LMBV-infected MsF cells, no apoptotic bodies were observed until 48 h p.i. (Figure 4A). Furthermore, the DNA content in LMBV-infected MsF cells and FHM cells was analyzed by flow cytometry. In LMBV-infected FHM cells, a prominent sub-G0/G1 peak representing detection of the apoptotic cells was observed and the percentage of the sub-G0/G1 phase increased from 37.3% at 24 h p.i. to 60.1% at 48 h p.i. However, LMBV infection induced changes in the cell populations at different phase of the cell cycle except the sub-G0/G1 phase in MsF cells. The cell percentage in the sub-G0/G1 phase was not significantly affected during LMBV infection compared to mock cells (Figure 4C,D). In order to determine the detailed characteristics of cell death induced by LMBV, DNA fragmentation was evaluated by TUNEL staining. As shown in Figure 5, the obvious TUNEL-positive cells were observed in LMBV-infected FHM cells. In contrast, even in the late stage of infection, few LMBV-infected MsF cells were positive for TUNEL staining. No TUNEL-positive signals were observed in both mock-infected MsF and FHM cells. Taken together, LMBV infection induced nonapoptotic cell death in MsF cells, which was cell-type dependent.

### 3.5. Caspase-3 and Caspase-8 Were Activated in LMBV-Infected MsF Cells

**Figure 6:**
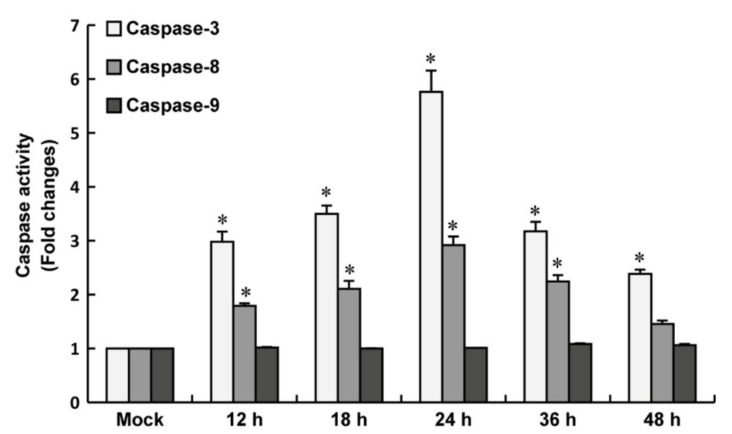
Caspase activation by LMBV infection in MsF cells. The activities of caspase-3, -8, and -9 were examined in LMBV-infected MsF cells using a caspase-3, caspase-8, and caspase-9 multiplex activity assay kit. * indicates *p* < 0.05.

Caspase is a family of cysteine proteases that play essential roles in apoptosis, necrosis, and inflammation [34]. Our previous study revealed that caspase-3, -8, and -9 were activated in LMBV-infected EPC cells [31]. To determine whether LMBV infection could activate caspase activation in MsF cells, the activities of different caspases, including caspase-3, -8, and -9, were examined using a caspase-3, caspase-8, and caspase-9 multiplex activity assay kit. As shown in Figure 6, the activities of caspase-3 and caspase-8 gradually increased from 12 h p.i. and reached a peak level of about 5.8-fold and 2.9-fold at 24 h p.i. in LMBV-infected MsF cells compared to that in mock-infected cells, respectively, and then their activity level gradually decreased. Differently, no significant increase in caspase-9 activation was detected during LMBV infection in MsF cells compared to mock cells.

### 3.6. LMBV Induced ΔΨm Depolarization and ROS Production

**Figure 7:**
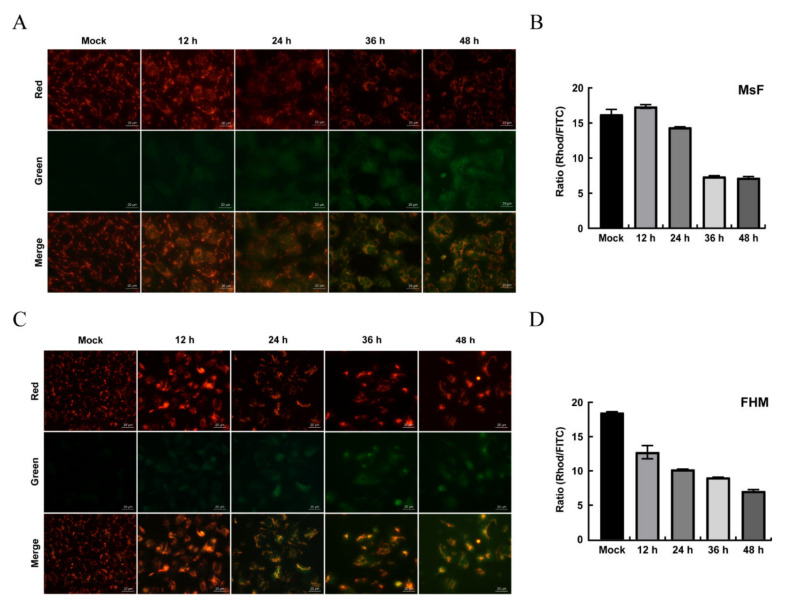
The changes in ΔΨm in LMBV-infected MsF and FHM cells. (**A**,**C**) Fluorescent microscopy observation of the changes in ΔΨm in LMBV-infected cells. (**B**,**D**) Quantitative analysis of the changes in ΔΨm in LMBV-infected cells by a microplate reader. After staining with JC-1, mock- or LMBV-infected cells were analyzed by fluorescence microscopy and a microplate reader.

The ultrastructure of LMBV-infected MsF cells showed that the morphology of mitochondria was altered (Figure 3B(i)), indicating that LMBV infection might affect mitochondrial function. JC-1, a lipophilic and cationic dye, selectively accumulates in mitochondria and reversibly changes the color from green to red as the membrane potential increases. To determine whether LMBV infection induced depolarization of the mitochondria, mock- and LMBV-infected MsF and FHM cells were stained with JC-1, and the alteration of ΔΨm was determined by the conversion from red fluorescence to green fluorescence. As shown in Figure 7, only bright red fluorescence was detected in mock cells. In virus-infected MsF cells, at 12 and 24 h p.i., bright red fluorescence was detected while limited green fluorescence was observed. The red fluorescence gradually weakened while the green fluorescence signals were enhanced in some virus-infected MsF cells at 36 h p.i. With the infection time, the minority of cells emitted red fluorescence and the number of cells emitting green fluorescence gradually increased at 48 h p.i. Consistently, the quantitative results show that the ratio of Rhod/FITC remained unchanged in LMBV-infected MsF cells 24 h after LMBV. However, the ratio of Rhod/FITC in LMBV-infected MsF cells significantly decreased from 16.2% up to 7.1% at 36 h (Figure 7B). At 12 h p.i., enhanced green fluorescence was observed in a few LMBV-infected FHM cells, accompanied by a decrease in red fluorescence. With the infection time, the number of cells emitting green fluorescence gradually increased. The ratio of Rhod/FITC decreased from 18.6% to 12.3% and 6.4% in LMBV-infected FHM cells at 12 h and 48 h, respectively. Thus, our results show that LMBV infection induced a disruption of ΔΨm in both MsF and FHM cells.

**Figure 8:**
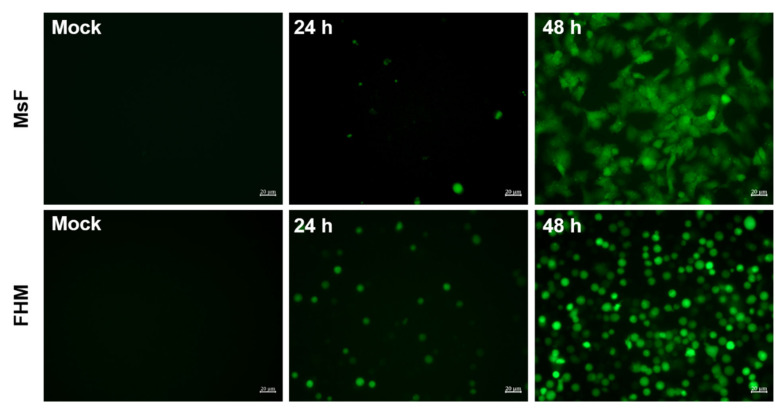
LMBV infection induced ROS generation in MsF cells or FHM cells. After staining with CM-H2DCFDA, mock- or LMBV-infected cells were observed under fluorescence microscopy.

Furthermore, the effect of LMBV infection in MsF cells on intracellular ROS was determined using a redox-sensitive fluorescence probe, CM-H2DCFDA, and then DCF fluorescence was observed under a fluorescent microscope. As shown in Figure 8, no green fluorescence signals were observed in mock-infected cells. Few cells labeled with green fluorescence were observed at 24 h p.i. while the number of cells emitting green fluorescence was obviously increased at 48 h p.i. (Figure 8). Differently, an increase in ROS production was detected in FHM cells during LMBV infection and this was time dependent.

## 4. Discussion

Largemouth bass (*Micropterus salmoides*) is one of the most important economic fishes for freshwater aquaculture. Iridovirus and rhabdovirus are two important viral pathogens of largemouth bass, which cause high mortality of juvenile and larval fish, respectively [28,35]. However, few cell lines originating from largemouth bass have been established until now, and limited literature has focused on the pathogenic mechanism of these viruses in host cells. Here, a new cell line, designated as MsF, was developed from the fin of largemouth bass, and its sensitivity to LMBV was evaluated. Our results revealed that MsF cells are susceptible to LMBV, as evidenced by the increase in viral gene transcription and viral titers. Furthermore, a large number of virus particles were observed in LMBV-infected MsF cells, indicating that LMBV replicated well in MsF cells. Thus, the established in vitro LMBV infection model will provide a suitable platform to study the LMBV pathogenesis in host cells.

It has been reported that iridoviruses assemble into assembly sites, also known as viral factories or viromatrix [36]. The microscopic and ultrastructural observations showed that two or more viral assembly sites were observed in LMBV-infected MsF cells. Similarly, two assembly sites could be observed in RGV-infected cells and African swine fever virus (ASFV)-infected cells [36,37]. Differently, our previous study showed that only one assembly site was observed in SGIV-infected cells [33]. In addition to virus particles assembled at different stages, the amorphous structures, including tubules and some membranous materials, were observed within the assembly sites in RGV-, ASFV-, and SGIV-infected cells [33,36]. Of note, tubular structures with an ~20-nm diameter were also observed within the assembly sites in LMBV-infected cells.

Upon virus infection, the manifestation of cell death by viruses or hosts determines the distinct consequences in the progression of viral pathogenesis, such as abortive, productive, and destructive infections. Increased evidence has demonstrated that virus infection evokes programmed cell death (PCD) in a cell-type-dependent fashion. For example, SGIV infection in GS cells induced nonapoptotic cell death while it evoked typical apoptosis in FHM, BM (barramundi muscle), and BSB (barramundi swim bladder) cells [24,25]. As a member of the genus *Ranavirus*, does LMBV also induce non-apoptotic cell death in host cells? To ascertain this point, we investigated the characteristics of cell death induced by LMBV in MsF cells. Interestingly, our results showed that LMBV infection induced obviously different CPE features in MsF and FHM cells, and no apoptotic bodies were observed in LMBV-infected MsF cells, suggesting that the cell death induced by LMBV infection in MsF cells was different from apoptosis. However, a large number of apoptotic bodies were observed in LMBV-infected FHM cells, similar to other non-host cells (EPC cells) in our previous study [31]. Consistently, other ranaviruses, including FV3, RGV, and EHNV, all evoked typical apoptosis after infection in non-host cells [19,20,21,22].

An increasing number of studies have demonstrated that caspases exert critical function both in apoptotic and non-apoptotic cell death [38]. Here, our results showed that caspase-3 and caspase-8 were activated in LMBV-infected MsF cells, and no significant increase in caspase-9 activation was detected during LMBV infection. Differently, the activities of caspase-3, -8, and -9 were increased in apoptotic cells induced by LMBV infection [31]. In general, the intrinsic apoptosis pathway is triggered when the mitochondrial integrity is impaired in response to stressors, such as DNA damage, endoplasmic reticulum stress, and ROS stress, and caspase-3 and caspase-9 were activated. The extrinsic apoptosis pathway is initiated through the binding of death ligands to their corresponding receptors, and caspase-8 and caspase-3 are activated successively [38]. In addition, caspase-8 was also found to be involved in pyroptosis induced by the cleavage of gasdermin (GSDM) family proteins [39]. Thus, caspase-8 has been considered as a key protein of crosstalk signaling for apoptosis, necroptosis, and pyroptosis [14,40]. Although caspase-8 and caspase-3 were significantly activated in LMBV-infected MsF cells, their detailed roles in this form of cell death need further investigation.

Mitochondria are highly dynamic organelles, and their structure and distribution are crucial for the cellular functions. In LMBV-infected MsF cells, we found that the cristae of mitochondria were faint or disappeared, and vacuolized mitochondria were observed around the assembly sites. Moreover, disruption of ΔΨm and ROS generation was detected in both LMBV-infected MsF cell and FHM cells. Differently, although ROS production was increased in both SGIV-infected host cells and non-host cells while disruption of ΔΨm was not detected in SGIV-infected host cells [24], suggesting that the types of cell death induced by these two ranaviruses are different. Except for apoptosis, ROS stress could induce multiple forms of cell death, including inflammasome-driven pyroptosis, necroptosis, ferroptosis, and autophagic cell death [41,42]. Recently, a highly interconnected PCD, termed PANoptosis, has been defined. PANoptosis is considered as an inflammatory PCD pathway regulated by the PANoptosome complex, which includes the mixture features of pyroptosis, apoptosis, and/or necroptosis [1]. Combined with our previous study, we propose that LMBV infection induces different forms of cell death in host and non-host cells. Although LMBV-infected MsF cells showed the characteristics of non-apoptosis cell death, the involved signal pathways might crosstalk and interconnect between apoptosis and other PCD during LMBV infection. It will be helpful to clarify the potential molecular mechanism underlying LMBV-induced non-apoptotic cell death in host cells in a further study.

## 5. Conclusions

In the present study, a new cell line, designated as MsF, was established from the fin of largemouth bass, and was applied to study the potential mechanism underlying LMBV infection-induced cell death in host cells. Our results showed that LMBV replicated well in MsF cells and induced non-apoptotic cell death, which was different from typical apoptosis in non-host cells. Moreover, only caspase-8 and caspase-3 were significantly activated in LMBV-infected MsF cells. In addition, disruption of ΔΨm and ROS generation was detected in LMBV-infected MsF cells. Thus, our data not only provides new evidence that ranavirus infection induces non-apoptotic cell death in host cells but also provides new insights regarding the mechanisms of LMBV pathogenesis.

## Figures and Tables

**Table 1:** The primers used in this study.

Primer Names	Sequence (5’-3’)
LMBV-MCP-F	CTCGCCACTTATGACAGCCTTGAC
LMBV-MCP-R	AACCCACGGGATAATGCTCTTTGAC
LMBV-MMP-F	GCGTATTTCGCACCCTCTG
LMBV-MMP-R	TAAGCGTCGCCCTTGTCTG
LMBV-DNMT-F	AATGTTTGGGTTTGAGGTAG
LMBV-DNMT-R	TCTTTAGCAGGCTGAGGG
MsF-β-actin-F	CCACCACAGCCGAGAGGGAA
MsF-β-actin-R	TCATGGTGGATGGGGCCAGG
FHM-β-actin-F	TACGAGCTGCCTGACGGACA
FHM-β-actin-R	GGCTGTGATCTCCTTCTGCA

## Data Availability

All datasets presented in this study are included in the article.
